# Photosynthetic recovery in drought‐rehydrated grapevines is associated with high demand from the sinks, maximizing the fruit‐oriented performance

**DOI:** 10.1111/tpj.16000

**Published:** 2022-10-28

**Authors:** Davide L. Patono, Daniel Said‐Pullicino, Leandro Eloi Alcatrāo, Andrea Firbus, Giorgio Ivaldi, Walter Chitarra, Alessandra Ferrandino, Davide Ricauda Aimonino, Luisella Celi, Giorgio Gambino, Irene Perrone, Claudio Lovisolo

**Affiliations:** ^1^ Department of Agricultural, Forest and Food Sciences University of Turin Grugliasco Italy; ^2^ Institute for Sustainable Plant Protection National Research Council Turin Italy; ^3^ Council for Agricultural Research and Economics‐Research Centre for Viticulture and Enology (CREA‐VE) Conegliano Italy

**Keywords:** water stress, drought, rehydration, ^13^C pulse‐chase technique, photosynthesis, respiration, sugar metabolism, sucrose synthase (*VvSusy*), cell wall invertase (*VvcwINV*), *Vitis vinifera* L

## Abstract

To understand how grapevine sinks compete with each other during water stress and subsequent rehydration, carbon (C) allocation patterns in drought‐rehydrated vines (REC) at the beginning of fruit ripening were compared with control vines maintained under drought (WS) or fully irrigated (WW). In the 30 days following rehydration, the quantity and distribution of newly fixed C between leaves, roots and fruits was evaluated through ^13^CO_2_ pulse‐labeling and stable isotope ratio mass spectrometry. REC plants diverted the same percentage of fixed C towards the berries as the WS plants, although the percentage was higher than that of WW plants. Net photosynthesis (measured simultaneously with root respiration in a multichamber system for analysis of gas exchange above‐ and below‐ground) was approximately two‐fold greater in REC compared to WS treatment, and comparable or even higher than in WW plants. Maximizing C assimilation and delivery in REC plants led to a significantly higher amount of newly fixed C compared to both control treatments, already 2 days after rehydration in root, and 2 days later in the berries, in line with the expression of genes responsible for sugar metabolism. In REC plants, the increase in C assimilation was able to support the requests of the sinks during fruit ripening, without affecting the reserves, as was the case in WS. These mechanisms clarify what is experienced in fruit crops, when occasional rain or irrigation events are more effective in determining sugar delivery towards fruits, rather than constant and satisfactory water availabilities.

## Significance Statement

In the rehydration phases following a period of drought, the strength of fruit carbon sinks inherited from the previous period of stress persists in grapevines, combined with a decisive photosynthetic recovery. These phases become the key moments in the life of fruit plants, especially if they coincide with the ripening phase of the fruit.

## INTRODUCTION

In temperate climate regions, rainfall is less evenly distributed during the growing season and the occurrence of prolonged periods of drought alternating with periods of abundant rainfall is increasing (Vilonen et al., 2022). In fleshy fruit crops, where the productivity and quality of fruits strictly depend on water availability, it is strategic to understand in more detail the dynamics of the plant response to alternations between low and high water availability (Ripoll et al., 2014).

The adaptation of grapevines to water deficit and recovery is a complex biological process (Herrera et al., 2022), where the most explored response mechanisms are linked to the hydraulic adaptation of the vine (Perrone et al., 2012), to stomatal regulation (Lavoie‐Lamoureux et al., 2017), and to their impact on photosynthesis (Galmés et al., 2007) and water use efficiency (Faralli et al., 2022). Decades of research have focused on water transport in the event of drought stress (Kuromori et al., 2022; Lovisolo et al., 2010), whereas the transport of carbon (C) in the plant and the related metabolic activities of roots and shoots are less studied (Douthe et al., 2018; Gambetta et al., 2020).

Within plants, C source–sink relationships affect photosynthate transport from sources towards other organs (sinks such as root tips, fruit and seeds) for further metabolism or storage. Currently, there is a change in the paradigm from a source‐limited model to a sink‐limited model, source activity (photosynthesis) depending on sink activity (tissue growth) (Fatichi et al., 2014; Körner, 2015). In recent years, it has been demonstrated that photosynthetic activity in plants experiencing water stress is not only affected by water transport, but also affected by the root C metabolism (Hasibeder et al., 2015). The first response of plants to the onset of water stress is the down‐regulation of root respiration that leads to a lower unloading rate of sucrose from the phloem in root. This decrease in the flow rate results in an accumulation of sucrose in the leaf leading to a feedback inhibition of photosynthesis. Similarly, the recovery of root metabolic activity with rehydration is immediate, thus resolving the imbalance between production and use (Hagedorn et al., 2016; Rodrigues et al., 2019).

Plants have different sinks competing with each other for photo‐assimilates, organized in a complex network (Knoblauch et al., 2016) that is based on a priority system, according to sink strengths (Ho, 2003), sink phenological phases and environmental stimuli (Wardlaw, 1990). Photosynthetic performance and relative availability of C fluctuate throughout the day, as do phloem loading and source–sink regulation, although it is not yet clear how phloem cells perceive sugar concentration and modulate signaling and expression of transporters [e.g. Sugar Will Eventually be Exported Transporters (SWEET) genes] (Chen et al., 2012; Keller et al., 2021). In conditions of prolonged stress, such as drought, plants activate different adaptation responses that strongly influence the mobilization and transport of C and, in turn, the source–sink performance (Lemoine et al., 2013).

By contrast to tree species, fruit crops such as grapevines introduce more complexity in the C sink relations: the root sink activity intersects berry growth and ripening that attracts large amounts of C during the growing season, in particular during fruit ripening (in grapevine, after *veraison*), competing strongly with the roots (Pastenes et al., 2014). This derives from an ancestral need to convey nutrients to the seed, with a parallel need to make the fruits palatable to the herbivore for seed dissemination that ensures the continuity of the progeny. Furthermore, the selection of the most productive phenotypes has made the fruit sink quantitatively competitive against the root sink (Ryan et al., 2018) to a greater extent than that observed in forest plants, where roots completely orchestrate the response to stress (Hagedorn et al., 2016).

In the present study, we aimed to explore whether the rehydration process could act as regulator of crop performance in an environment with low water availability and a highly‐evapotranspirative atmosphere. It could be hypothesized that, in crop fruit plants, which show an increasing fruit sink strength from flowering to harvest, the root carries out its sink activity secondary to the fruit. The object of our research is to understand when, how much and how the root and fruit compete with each other, and if, during water stress and/or during the subsequent rehydration, the competition can be accentuated. To this end, we have conducted analyses through (i) the assessment of C allocation kinetics in the different plant sinks (root‐shoot‐fruit) competing in drought and post‐drought rehydrated vines; (ii) the measurement of the ecophysiological performances in root and shoot; and (iii) the analysis of transcripts of key genes involved in source–sink inter‐relationships. Carbon allocation patterns between different sinks can be adequately studied by means of ^13^C pulse‐labeling approaches in which temporal changes in the ^13^C isotope content of different plant parts after labeling with ^13^C enriched CO_2_ (^13^CO_2_) can be used to trace the distribution of neo‐photosynthates and follow C partitioning between sinks (Epron et al., 2012).

## RESULTS

### Water treatments and gas exchange analysis

**Figure 1:**
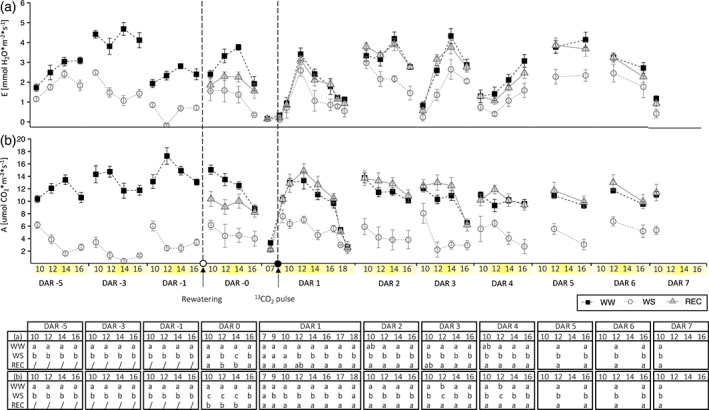
Whole‐plant gas exchange analysis. (a) Transpiration, *E* and (b) net photosynthesis, *A*. From DAR −5 to DAR −1, data for WW (black squares, *n* = 4) and WS (white circles, *n* = 8) plants before the rehydration event are represented; from DAR 0 to DAR 7, data for WW (*n* = 4), WS (*n* = 4) and REC (gray triangles, *n* = 4) plants after rehydration are plotted. Dotted vertical lines before and after DAR 0 show rehydration and ^13^CO_2_ pulse‐labeling. Statistical analysis of data was performed using one‐way anova followed by a *post hoc* Tukey's test. Lowecase letters in the table denote a statistically significant difference (*P* < 0.05).

**Figure 2:**
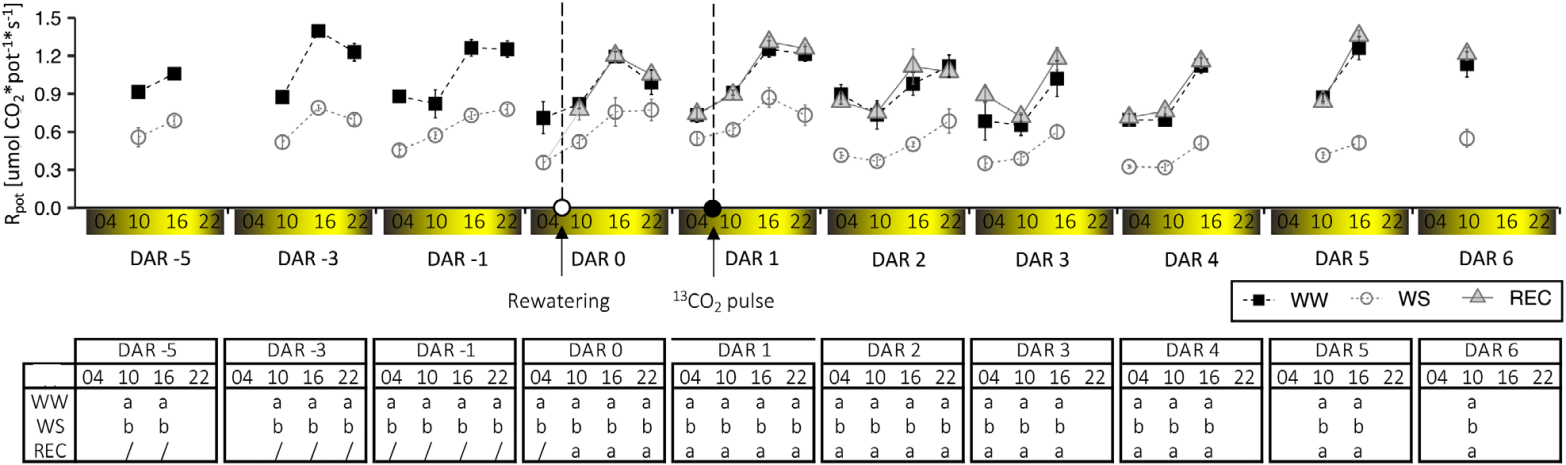
Whole‐plant gas exchange analysis. Below‐ground respiration (*R*_bg_). Symbols, replicates (*n*) and statistical analysis are as in Figure 1.

From the beginning of March, 3‐year‐old plants of grapevine *cv* Barbera with similar root volume were grown in pots and fully‐irrigated (WW) to prevent water stress. At the end of July, two‐thirds of the plants were exposed to water stress (WS) by drastically reducing the irrigation regime. On 20 August [i.e. the day after rehydration zero (DAR 0)], one‐half of the plants were rehydrated (REC) to pre‐stress conditions, whereas the other half were maintained under water stress. A whole plant gas exchange analysis in a custom‐built multichamber system was started 1 week before rehydration. WW plants maintained a higher transpiration of the whole canopy (*E*) (Figure 1a), net CO_2_ assimilation of the whole canopy (*A*) (Figure 1b) and below‐ground respiration (*R*_bg_) (Figure 2) than WS plants. Although *R*_bg_ of REC plants was rapidly restored to the level of WW plants within the first 2 h after rehydration, the levels of *E* and *A* of REC plants reached those of WW plants after 8 h. During the subsequent days, *R*_bg_ and *E* of REC plants were comparable to those of WW plants. *A* of REC plants was similar to that of WW plants during DAR 1 and 2, with a trend of up‐regulation in the central hours of the day that became statistically significant at DAR 3 and 4. From DAR 5 onwards, no differences in *A* between REC and WW plants were appreciable.

### Carbon allocation patterns to the different sinks following rehydration

**Figure 3:**
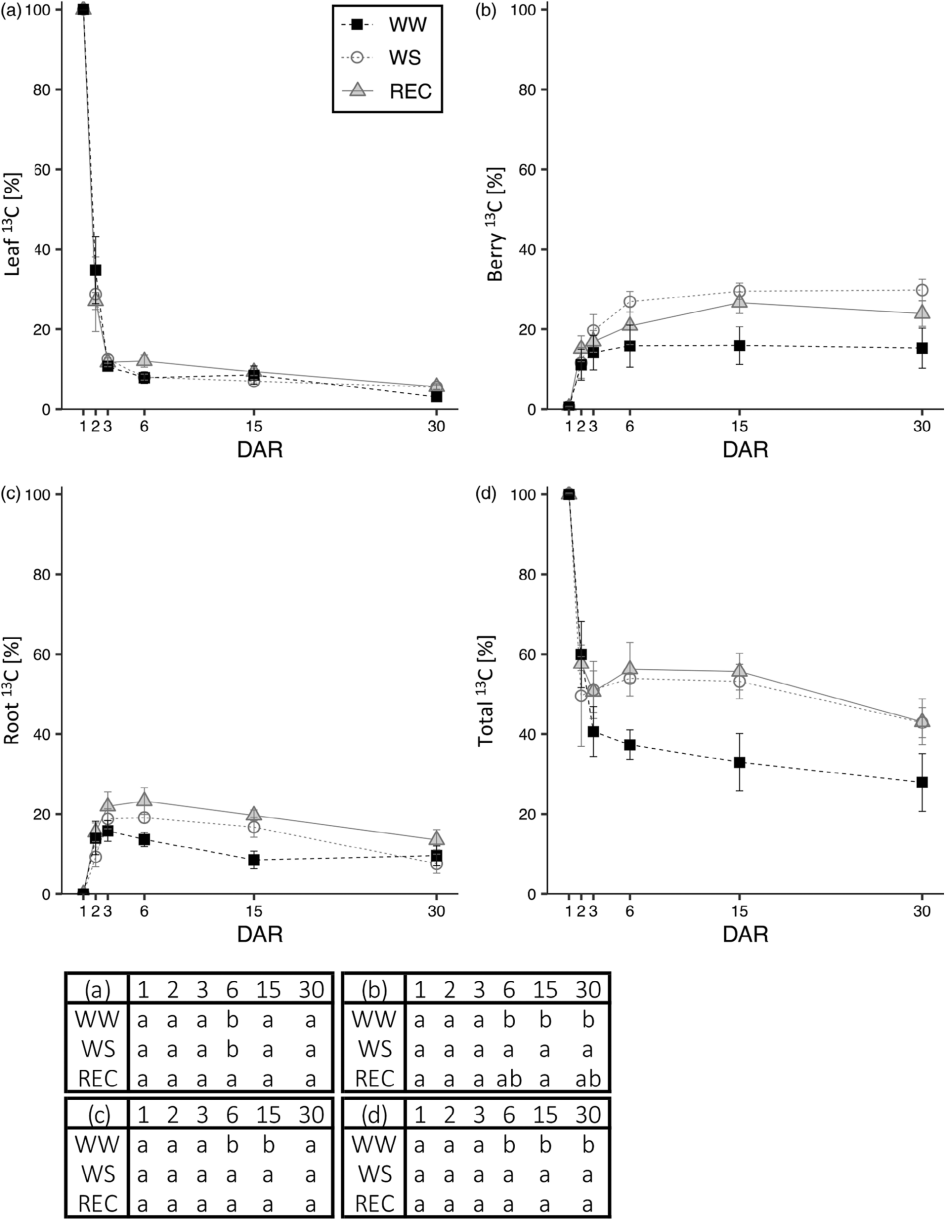
^13^C partitioning of neo‐photosynthates after a rehydration event. Partitioning of assimilated labeled ^13^CO_2_ during a feeding event at DAR 1. (a), (b), (c) and (d) represent % of labeled ^13^C at DAR 1, 2, 3, 6, 15 and 30, respectively, in leaf, berry, root and whole plant. Values are the mean ± SE (*n* = 3). Statistical analysis of data was performed using one‐way anova followed by a *post hoc* Tukey's test. Lowercase letters in the table denote a statistically significant difference (*P* < 0.05).

From 08.00 h to 12.00 h of DAR 1, nine plants (three for each treatment) were ^13^CO_2_ pulse‐labeled under climate‐controlled conditions (Figure S1). Total ^13^CO_2_ fixed by each plant immediately after labeling (DAR 1) corresponded to 9.2 ± 3.4 mmol ^13^C plant^−1^ that was found exclusively in the leaves, without significant differences among treatments. Up to 90% of this pool of newly assimilated C was rapidly lost from the leaves by respiration and reallocation to other plant parts within 2 days from labeling (DAR 3), irrespective of the irrigation regime. WW and WS leaves showed a slight further reduction of the residual ^13^C at DAR 6, although no further significant loss of C was observable at DAR 15 and DAR 30. REC plants did not lose ^13^C from leaves between DAR 3 and 6 but showed a decrease thereafter. By DAR 30, all plants showed the same residual amount of ^13^C in the leaves that amounted to approximately 5–8% of assimilated C (Figure 3a).

Allocation of ^13^C to the berries increased with time over the first days after labeling and subsequently reached a stable amount by DAR 6 to 15 (between 16 and 30% of fixed ^13^C) with no significant subsequent loss of ^13^C. However, although the maximum proportion of ^13^C was reached within DAR 6 in WW and WS plants, ^13^C allocation to the berries of REC plants continued to increase until DAR 15. The final proportion of ^13^C allocated to the berries was higher in WS and REC plants compared to WW ones (Figure 3b).

By contrast, ^13^C allocation to the roots increased to a maximum by DAR 3 in WW, whereas, in WS and REC, root C allocation continued to increase slightly between DAR 3 and DAR 6. Subsequently, WW plants quickly lost ^13^C between DAR 3 and 15 and all the ^13^C remaining at DAR 15 persisted also at DAR 30. On the other hand, WS and REC plants showed a slower loss of ^13^C that persisted also between DAR 15 and 30. By the end of the experiment, the residual proportion of fixed ^13^C in the roots of WW, WS and REC plants (approximately 10%) was not significantly different (Figure 3c).

Considering the total amount of fixed ^13^C in the different pools, there was a strong decrease in fixed ^13^C in the first 3 days that continued to decrease faster in WW plants with respect to WS and REC plants, resulting in a final proportion of fixed ^13^C of approximately 40% for WS and REC plants and 30% for WW plants (Figure 3d).

**Figure 4:**
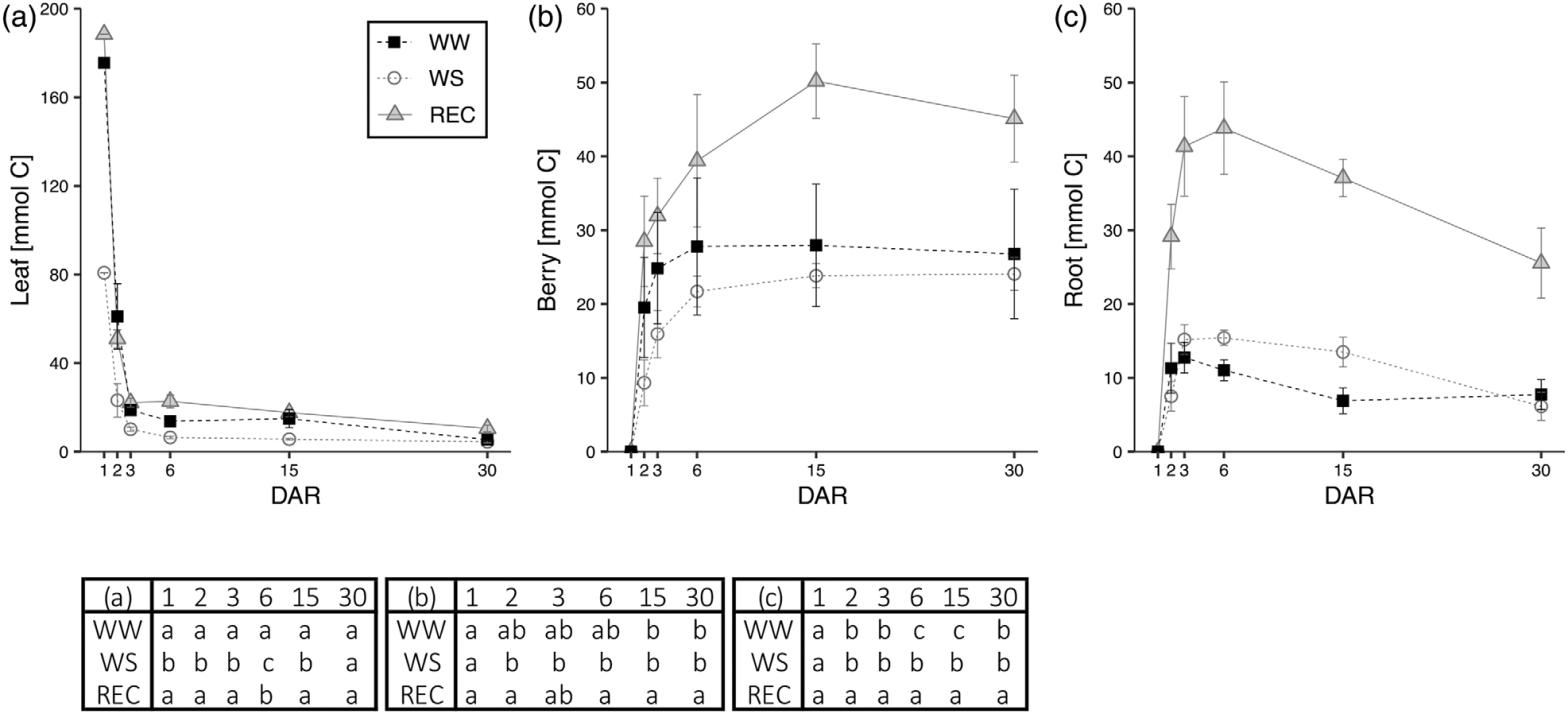
Carbon accumulation. Amounts of carbon allocations in different sinks obtained by multiplying the residual ^13^C percent found in the different pools at DAR 1, 2, 3, 6, 15 and 30 with the integrated daily *A* of DAR 1 (the ^13^C pulse day) in the leaf canopy (a), in all berries (b) and in the whole root (c). Values are the mean ± SE (*n* = 3). Statistical analysis of data was performed using one‐way anova followed by a *post hoc* Tukey's test. Lowercase letters in the table denote statistically significant variations (*P* < 0.05).

Considering that the different irrigation treatments affected both net photosynthesis as well as the partitioning of newly assimilated C between the different sinks, we estimated the amount of C transferred to the sinks following rehydration (DAR 0) by coupling daily integrals of A and total respiration (*R*_tot_ = *R*_bg_ + *R*_cd_) at DAR 1 with the allocation of ^13^C fixed at DAR 1 to the different sinks. In detail, plant gas exchange outputs at DAR 1 were integrated over 24 h, and total daily *A*, *R*_bg_, the respiration of the whole‐canopy during dark hours (*R*_cd_) and *R*_tot_ are reported in Table 1, showing that the ratio between *R*_tot_ and *A* was significantly higher in WS plants than in WW and REC plants. We calculated the residual amount of C allocated to the different pools after sink respiration and/or re‐mobilization by multiplying the proportion of residual ^13^C in the different pools at DAR 1, 2, 3, 6, 15 and 30, with the integrated daily *A* of DAR 1 (the ^13^C pulse day). Figure 4 reports this information and shows how total C allocated to berry and root was similar between WS and WW plants and higher in REC plants. Already from DAR 2 in root, and 2 days later in fruit, the amount of C in the REC treatment was significantly higher than in the control plants (both WW and WS). The WS plants allocated more C below‐ground than WW controls in the first 15 days, but then the consumption (respiration or translocation) brought the assimilated C to a level comparable to that of WW controls. Also in the REC roots, the maximum amount of C at DAR 6 tended to drop, indicating consumption and/or reallocation but the total amount of assimilated C that remained in the root at DAR 30 was significantly higher than WW and WS plants. By contrast, C accumulation in the berries of REC plants remained stable and constant in time (Figure 4).

**Table 1:** Gas exchange integrals at DAR 1

	Daily *A*	Daily *R*_bg_	Daily *R*_cd_	Daily *R*_tot_	*R*_tot_/*A*
	(mmol CO_2_)	(mmol CO_2_)	(mmol CO_2_)	(mmol CO_2_)	(%)
WW	176 ± 14 a	92 ± 10 a	9 ± 1 a	101 ± 9 a	57 ± 2 b
WS	81 ± 10 b	59 ± 5 b	8 ± 1 a	67 ± 6 b	83 ± 4 a
REC	188 ± 33 a	90 ± 2 a	9 ± 1 a	100 ± 2 a	54 ± 8 b

Daily integrals of: whole‐canopy assimilation (*A*), respiration of the whole‐canopy during dark hours (*R*_cd_), below‐ground respiration (*R*_bg_), total respiration (*R*_tot_ = *R*_bg_ + *R*_cd_) at DAR 1. Values are the mean ± SE (*n* = 4). Statistical analysis of data was performed using one‐way anova followed by a *post hoc* Tukey's test. Lowercase letters denote a statistically significant difference (*P* < 0.05).

**Figure 5:**
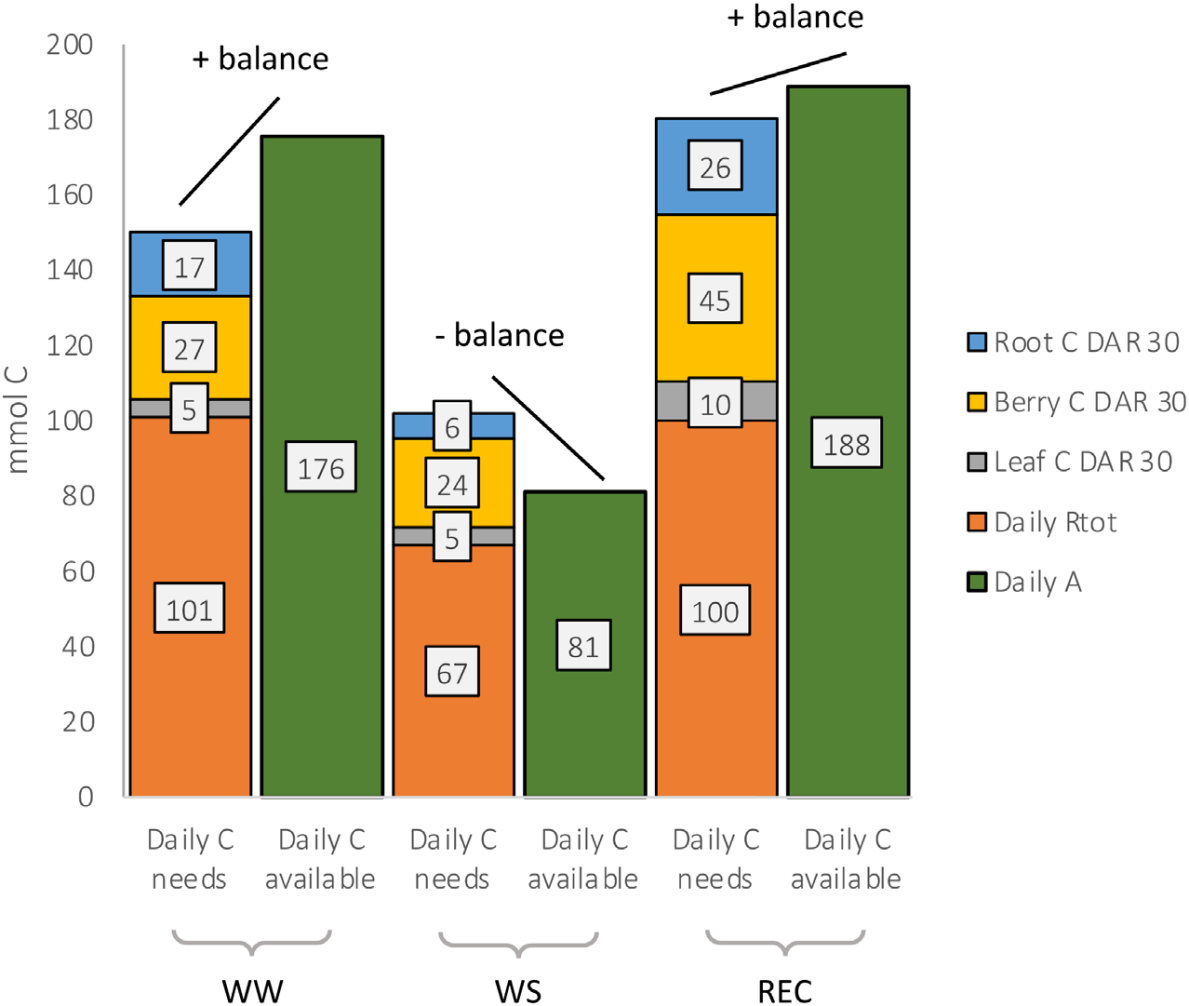
Model of C allocation to different C pools. The model is the combination of data from gas exchange analysis with data from pulse‐chasing C isotope analysis. In blue, yellow and gray, we show the amount of neo‐photosynthates that will be permanently stocked respectively in root, berries and leaf C pools. In orange and green, daily *R*_tot_ and daily *A* is reported.

The amount of assimilated C that remained in the leaf at DAR 30 was much lower compared to the other two C pools, although nonetheless slightly higher in REC plants compared to WS and WW plants. Adding the total amounts of newly fixed C remaining in the roots, berries and leaves at DAR 30 to the daily *R*_tot_, we observed that the amount of daily C assimilated was sufficient to support C accumulation and root and shoot respiration in WW and REC plants, but, in contrast, not sufficient in WS plants (Figure 5).

The accumulation levels of fresh and dry matter, measured at DAR 30 in berries, reflected the carbon fluxes described so far and the levels of water potential measured. The REC grapes generally showed higher levels than the WW ones, in turn higher than the WS ones (Table 2).

**Table 2:** Weight of the berry (g), production of grapes per plant (kg), number of berries per plant (#), degree Brix (°Brix) and total acidity as tartaric acid (g L^−^1) measured on DAR 30

	Weight of the berry	Production of grapes per plant	Number of berries per plant	°Brix	Total acidity as tartaric acid
	(g)	(kg)			(g L^–1^)
WW	1.80 ± 0.14 b	0.34 ± 0.02 b	192 ± 15 a	24.5 ± 0.46 b	8.00 ± 0.35 a
WS	1.53 ± 0.09 c	0.29 ± 0.05 c	192 ± 33 a	23.8 ± 0.63 b	6.65 ± 0.66 a
REC	2.27 ± 0.04 a	0.47 ± 0.04 a	205 ± 36 a	25.6 ± 0.36 a	7.25 ± 0.43 a

Values are the mean ± SE (*n* = 4). Statistical analysis of data was performed using one‐way anova followed by a *post hoc* Tukey's test. Lowercase letters denote statistically significant variations (*P* < 0.05).

### Transcript expression analyses of key genes involved in source–sink inter‐relationships

**Figure 6:**
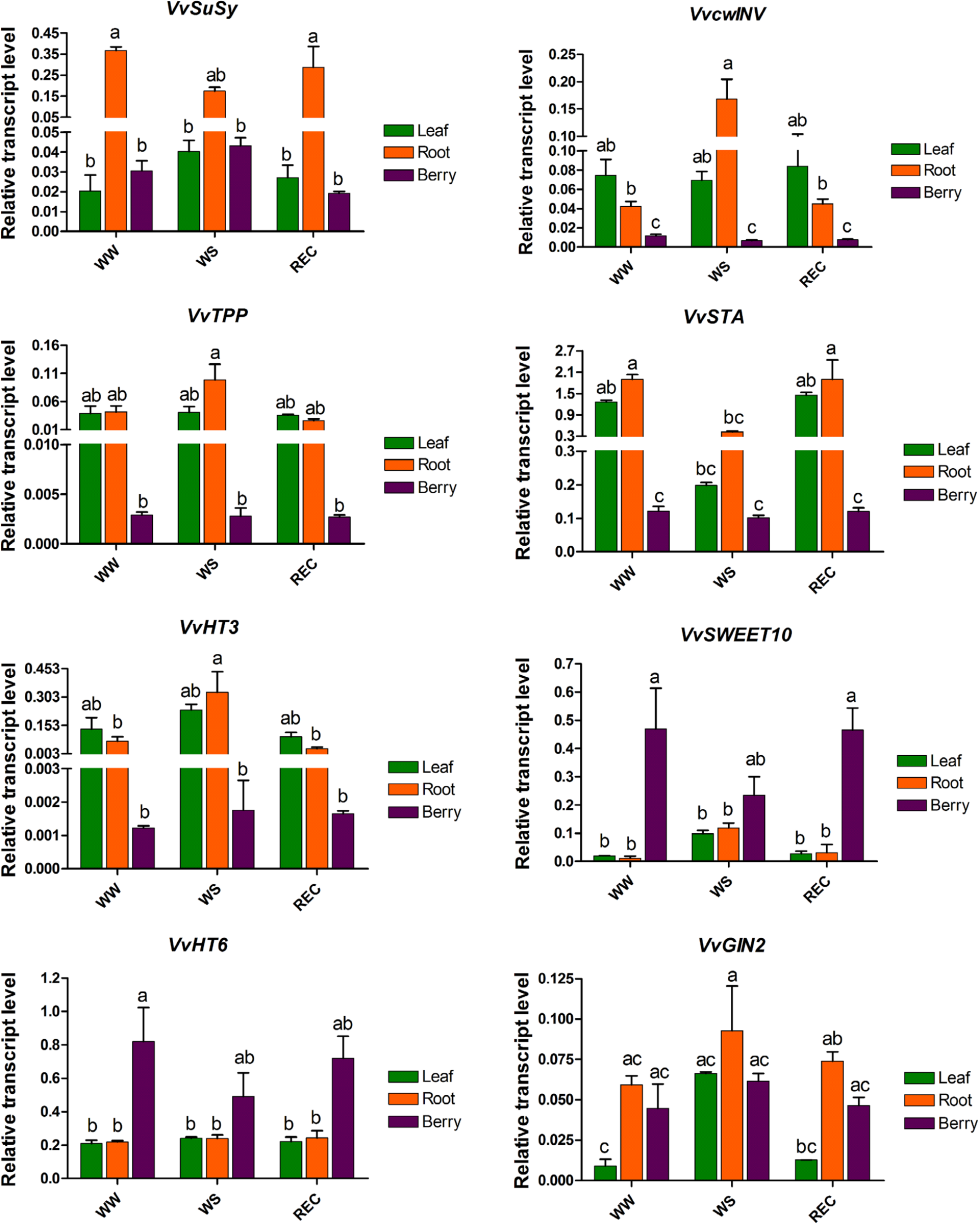
Transcripts of key genes of sugar metabolism. Relative expression level of sucrose synthase (*VvSusy*), cell wall invertase (*VvcwINV*), threalose 6‐phosphate phosphatase (*VvTPP*), starch synthase (*VvSTA*), hexose transporter 3 (*VvHT3*), Sugar Will Eventually be Exported Transporter 10 (*VvSWEET10*), hexose transporter 6 (*VvHT6*) and vacuolar invertase 2 (*VvGIN2*) genes in leaf, root and berry tissues sampled from WW, WS and REC plants at DAR 4, as determined by qRT‐PCR signals normalized to actin (*VvACT*) and ubiquitin (*VvUBI*) transcripts. Data are presented as the mean ± SE of three biological and technical replicates. Gene IDs and oligonucleotides used for each gene are indicated in Table S1. Different lowercase letters above the bars indicate a significant difference according to a *post hoc* Tukey's test (*P* ≤ 0.05).

The expression of different carbohydrate metabolism‐related genes was analyzed in source and sink tissues of WW, WS and REC plants over a time course characterizing the early phases after rewatering (DAR 0, DAR 1 and DAR4), aiming to add information at the molecular level about carbon allocation dynamics (for DAR 0 and DAR 1, see Figures S2; for DAR 4, see Figure 6). In general, the gene expression trends were similar during the early phases considered, thus we decided to describe more in detail the results occurring at DAR4 when ecophysiological measurements confirmed a fully recovery of REC plants (Figure 6). The sucrose synthase gene *VvSuSy* was expressed mainly in root and characterized by a lower expression level in WS treatment. An alternative route for sucrose breakdown in WS root was offered by the increased expression of the cell wall invertase (*VvcwINV*) gene, for which the expression trend was generally complementary to that of *VvSuSy* in WW, WS and REC root samples. Interestingly, WS root showed an increased expression of threalose 6‐phosphate phosphatase (*VvTPP*) gene, which is responsible for the synthesis of threalose from the precursor threalose 6‐phosphate. The availability of new photosynthates after rehydration allowed the REC root to increase the starch synthesis (*VvSTA*, starch synthase) mirroring the WW root behavior, whereas, in WS root, *VvSTA* did not increase the expression level. This result agrees with the high hexose mobilization confirmed by the increased expression level of the hexose transporter 3 (*VvHT3*) in WS root compared to the same tissue of WW and REC plants. In berry, two transcripts among the genes analyzed showed high expression, mainly in WW and REC plants: the Sugar Will Eventually be Exported Transporter 10 (*VvSWEET10*), which is responsible for phloem unloading in sink tissues, and the vacuolar hexose transporter 6 (*VvHT6*), driving the carbohydrates to storage in the vacuole. Similarly, the vacuolar invertase *VvGIN2* showed expression in the berry, reinforcing the sucrose compartmentalization in the vacuole (Figure 6).

## DISCUSSION

### Carbon balance in drought‐rehydrated ripening grapevines

In the present study, droughted vines rehydrated at *veraison* were pulse‐labeled with ^13^CO_2_ together with other vines maintained in water deficit or fully irrigated. The ^13^C absorbed by the leaves with photosynthesis during labeling was subsequently used to trace the phloem flows of newly assimilated C towards the strongest sinks in the 30 days of the post‐*veraison* phase, when the ripening processes of the grape occurred triggering C allocation towards the sinks. From the combined analysis of the ^13^C allocation patterns, as well as photosynthesis and respiration gas exchanges of the plant (shoot and root) and rhizosphere compartments, we were able to demonstrate several inter‐relationships occurring among plant organs during a rehydration event following a drought period either above‐ or below‐ground.

The resumption of root metabolic activity and post‐rehydration photosynthesis is almost immediate (a few hours and less than 24 h, respectively), showing how adapted the vine is to tolerating water stress. By contrast, beech, a mesophilic plant not adapted to arid climates (Fotelli et al., 2001), has been shown to take a few weeks for photosynthesis to recover to pre‐stress conditions (Hagedorn et al., 2016), Furthermore, the presence in grapeveines of the fruit sink with considerable strength, triggered a photosynthetic and respiratory energy demand (Fatichi et al., 2014; Körner, 2015).

The water regime strongly influenced the partitioning of C towards the different sinks. Water stress caused a greater allocation of the newly photosynthesized carbonaceous resources to the berry (approximately double compared to WW controls), which are stored in a stable manner. On the other hand, the C allocated below‐ground over 30 days is mostly consumed. The plant in recovery diverts the same percentage of labeled C to the berry as the plants in water stress, although, in absolute amounts, its photosynthesis is approximately double that under water stress (it is comparable or even higher than photosynthesis in WW control plants). Therefore, the total C allocated to the berry is approximately 50% higher in recovery than in the irrigated control. These physiological mechanisms are the basis of what is often experienced in irrigated fleshy fruit crops, where it has been previously shown that occasional irrigation events are more effective in determining sugar‐related production, rather than maintaining a constant satisfactory water state (Chaves et al., 2010). Moreover, the rain fed areas with viticultural vocation present microclimatic situations of summer aridity with only occasional rains (Charrier et al., 2018).

Through a daily respired/photosynthesized C balance, we show that during the ripening of the berry (30 days post‐*veraison*) 57% of the C assimilated in the irrigated condition is respired. In the same period, the accumulation of neo‐photosynthates is approximately 28%, showing that plant photosynthesis can support C accumulation in sinks without affecting plant reserves accumulated pre‐*veraison*, as shown by Rossouw et al. (2017) in irrigated grapevines. By contrast, upon water stress, 83% of the daily C assimilated is respired. Because 43% of neo‐photosynthesized C is stored in a stable manner, we conclude that the plant should affect C radical reserves accumulated before *veraison* to support the respiration rate. After rehydration in REC pants, 54% of the daily C assimilated of the post‐*veraison* month is respired, similarly to that occurring in WW controls; approximately 43% of neo‐photosynthesized C is stored in a stable manner (especially in berries), and much more compared to under the WW condition. However, the increase in A was able to support the requests of the sinks, without affecting the reserves, as was the case in WS. During WS, the lack of turgor acting as major limitation to growth (Hernandez‐Santana et al., 2021) forced plants to affect C reserves, adding evidence to the sink limitation hypothesis to photosynthesis (Fatichi et al., 2014). The highest proportion of photosynthates was partitioned into fruits (berries) and, in WS plants, it was almost double than that under the WW condition, as indicated in Figure 5 (for fruits: daily C needs/daily C available: 27/176 = 15% in WW plants, 24/81 = 29% in WS plants and 45/188 = 23% in REC ones). From a wider point of view, this indicates why fruits are usually seen as stronger sinks than other organs or even why fruit growth is generally seen as less sensitive to water stress than vegetative growth.

### Molecular evidence supporting the model

Delivery of labeled ^13^C to the different sinks was observed in parallel with the expression of genes involved in carbohydrate metabolism. Genes encoding proteins that affect the delivery of sucrose to the sinks and which catalyze the hydrolysis of the sucrose discharged to trigger respiration or carbon storage have been analyzed. SuSy is an enzyme with a central role in the source–sink coordination, and it catalyzes the breakdown of sucrose in sink tissues to maintain the concentration and pressure gradient operational in the phloem (Gessler, 2021). The *SuSy* gene was expressed mainly in roots. In rehydrated roots, as a result of the availability of new photo‐assimilated resources and the recovery of root respiration, the molecular machinery quickly adjusted to that of WW plants, whereas WS root showed a lower level of *SuSy* expression, probably to compensate for the lack of assimilation. Interestingly, *cwINV* gene expression was significantly higher in WS root compared to WW and REC roots, ensuring an alternative route of sucrose breakdown and maintenance of the root sink strength also in the water stress condition, as confirmed by the ^13^C partitioning analysis. The relative impacts of SuSy and invertase on C allocation appear to be dependent on tissue, species, developmental stage and season (Dominguez et al., 2021). Moreover, popular RNA interference transgenic lines for *SuSy* showed increased invertase activity, suggesting a partial compensation of the two enzymes in the sucrose cleavage activity, a phenomenon that we can retrieve in our data also by looking at the complementary expression of *SuSy* and *cwINV* transcripts in the WW, WS and REC roots. The understanding of why the grapevine root leans towards preferential expression of invertase during water stress requires further experiments. It is known that both pathways degrade sucrose but the products of their reactions differ considerably; the literature suggests that, although SuSy could be involved in increased biomass (Gessler, 2021; Xu et al., 2012), invertases could have a greater ability to stimulate specific sugar sensors (Ruan, 2012; Ruan et al., 2010). The involvement of the water stressed root in sucrose signaling was confirmed by the overexpression of the *threalose 6‐phospate phosphatase* (TPP) transcript, catalyzing the second step of threalose synthesis. Trehalose accumulation confers high tolerance levels to different abiotic stresses (Garg et al., 2002) and, together with the precursor threalose 6‐phosphate, plays a key role in carbon allocation and stress responses in plants (Morabito et al., 2021). We could speculate that, through sugar signaling, the WS root orchestrated maintenance of the sink strength despite the unfavorable conditions for C allocation. Hexoses produced from sucrose cleavage were not used for starch synthesis in WS root, as suggested by the low expression of *VvSTA* and the high expression of the *HT3*, confirming the mobilization of hexoses. By contrast, the REC root started the starch synthesis with a quick adjustment to the WW condition.

Sugar Will Eventually be Exported Transporters (SWEETs) 10 is a plasma membrane sucrose transporter of clade III SWEETs involved in phloem unloading (Eom et al., 2015; Savoi et al., 2021). It is one of the two transcripts in our experiment expressed at a high level in the berry. Although the main driver of sucrose unloading in the berry was the developmental stage (*veraison*), as suggested from the high *SWEET10* expression level over all the time course, a slight treatment effect could be noted. As a result of photosynthesis and assimilation recovery, the REC plant was able to maximize the C allocation in the fruit. Interestingly, because the *SWEET10* transcript level remained low in the REC root tissue, we can suggest that the prompt increase in root respiration after rehydration was not accompanied by an increase in the unloading rate of sucrose in root, in contrast to that occurring in non‐fruit trees (Hagedorn et al., 2016). In grapevine, when the fruit is present, our experiment suggests that the root becomes a secondary sink. The unloading of sucrose was guaranteed by the *SWEET10* expression also in WS berries, although to a minor extent probably because of the limited photosynthates available in the stress condition diverted towards the root, as confirmed by *SWEET10* expression increasing in this tissue compared to WW and REC plants. Finally, analysis of the *vacuolar hexose transporter HT6* expression level, which is the second most highly expressed gene in berry, pointed out that this transporter allowed the hexoses accumulation in the vacuole, so that the sink strength can be maintained to attract more C (as Susy does in root). Moreover, the storage of sucrose in the vacuole was driven also by the *vacuolar invertase GIN2*. In general, berry metabolism appeared to be stopped as confirmed by the generally low expression level of carbohydrate metabolism‐related genes analyzed, with the exception of the genes described above that are key modulators for hexose and sucrose accumulation and cell expansion (Ruan et al., 2010) in the phenological stage of *veraison*. This molecular difference underlines what has been seen in our C distribution model between the root and fruit sinks, which shows how the allocated C amount remains constant in the REC fruit over 30 days, with no redistributive decreasing trend, as in the root.

### Possible implications of the research

Confirming the measurements of carbon fluxes and water potential levels that plants experienced during the experiment, berries of the REC plants were found to be the heaviest and to demonstrate the highest sugar concentration at DAR 30. In WS plants, the low growth levels of the berries that developed in a context of scarce water availability were not combined with low levels of sugar concentration [expressed in degree Brix (°Brix)], found significantly not lower than in the WW berries, confirming what is shown as carbon accumulation in Figure 4b.

Our experimental design mimicked a peculiar scenario, optimized to observe by how much as well as how root and fruit compete with each other, but not necessarily aligned with other possible scenarios in the field. It combines with a field situation, where, until *veraison*, grapevines mainly perceive a water deficiency, followed by rain fed into the subsequent phases of the productive cycle. In this scenario, an increase of C allocation in berries positively affects berry quality in relation not only to the accumulation of primary metabolites *per se*, but also to the accumulation of secondary metabolites as glycosides in the cell vacuoles (Ferrandino & Lovisolo, 2014). Furthermore, C and sugar biosynthesis‐transport related genes combine with the activation of the phenylalanine ammonia lyase (PAL), the key enzyme of the phenylpropanoid pathway (Pirie & Mullins, 1976).

However, in some viticultural areas, pre‐*veraison* water deficits could be less frequent than water deficits later in the ripening process. Scholasch and Rienth (2019) reviewed water deficit‐mediated changes in vine and berry physiology, highlighting how this latter scenario, opposite to that described in our experimental setup, could increase berry quality. This is because reducing water availability after *veraison* positively affects yield components via both a reduction of berry volume (Zúñiga et al., 2018) and the activation of ABA‐related biosynthetic pathways (Ferrandino & Lovisolo, 2014). As a specular confirmation, Intrigliolo et al. (2016) showed that a post‐*veraison* irrigation results in a 26–30% yield increase compared to rain fed vineyards that experienced a post‐*veraison* water deficit.

In the present study, the effects of the carbon distribution wave following rehydration combined with the expected delivery of phloem‐water. The genotype we used (‘Barbera’ on ‘420A’) should mitigate the effects of a distinctly anisohydric response to water stress of the scion through the use of a rootstock that is not tolerant to drought, and therefore not inclined to force lowering of the water potential during drought (Lavoie‐Lamoureux et al., 2017; Tramontini et al., 2013). In cases of varieties showing an anisohydric behavior grafted on tolerant rootstocks (e.g. descendants of *Vitis rupestris* L.), rehydration could have even more significant effects on the distribution wave of photosynthates; this is because the ability to compensate for the mechanisms of lowering the water potential (including osmotic adjustment, embolism repair and aquaporin overexpression; Lovisolo, Tramontini, et al., 2008) in stressful situations would allow these phenotypes a fast and active post‐rehydration recovery (Lovisolo et al., 2010; Scholasch & Rienth, 2019). By contrast, we can speculate that rehydration could be less effective in scions showing isohydric response to water deficit and/or rootstocks sensitive to drought (e.g. descendants of *Vitis riparia* L.) (Lovisolo, Tramontini, et al., 2008).

## CONCLUSIONS

Our results show how periods of water stress activate a molecular response in the plant C sinks to compensate for the reduction in photosynthetic C assimilation. In fruit crops, the fruits compete strongly with the root. This derives from an ancestral need to convey nutrients to the seed with a parallel need to make the fruits palatable to the herbivore for a seed dissemination that ensures the continuity of the progeny. Furthermore, the selection of the most productive phenotypes has made the fruit sink quantitatively competitive against the root sink, which is much more than that occurring in forest plants, where the root completely orchestrates the response to stress. In the rehydration phases, the strength of the sink persists but is combined with a photosynthetic recovery activated by the phloem downloading capacity directed towards the strongest C‐requesting sinks. This is so effective that the assimilation values of the rehydrated plants exceed those of the irrigated plants. In rehydration moments, the effects of maximum C assimilation and relative delivery to the requesting sinks take place. They therefore represent the key moments in the life of the fleshy fruit plants, especially if they coincide with the ripening phase of the fruit, as in our experimental design.

## EXPERIMENTAL PROCEDURES

### Plant material, growth condition and water stress treatment

Plants of *Vitis vinifera cv* Barbera grafted onto *Vitis riparia* × *Vitis berlandieri* 420A rootstocks were grown for 3 years in a 70‐L pot. In February, vines were taken out from their growing pots, the soil was removed and 24 plants with similar root volume were selected. Twelve selected vines were placed in custom metal pots (internal diameter 450 mm; depth 450 mm) with an air tight lid (for simultaneous measurement of *R*_bg_ and whole plant gas exchange, Figure S3), whereas another 12 were transferred to 70‐L plastic pots filled with 60 L of a 3:2 v/v sand‐peat mixture and 9 g of grapevine granular fertilizer (12 + 12 + 17 + 2 MgO + 20 SO_3_). Once the vines started to break dormancy, four shoots bearing a cluster were selected in each plant and, at the beginning of July, plant canopies were green‐pruned to a similar leaf area (LA) (approximately 0,5 m^2^).

**Figure 7:**
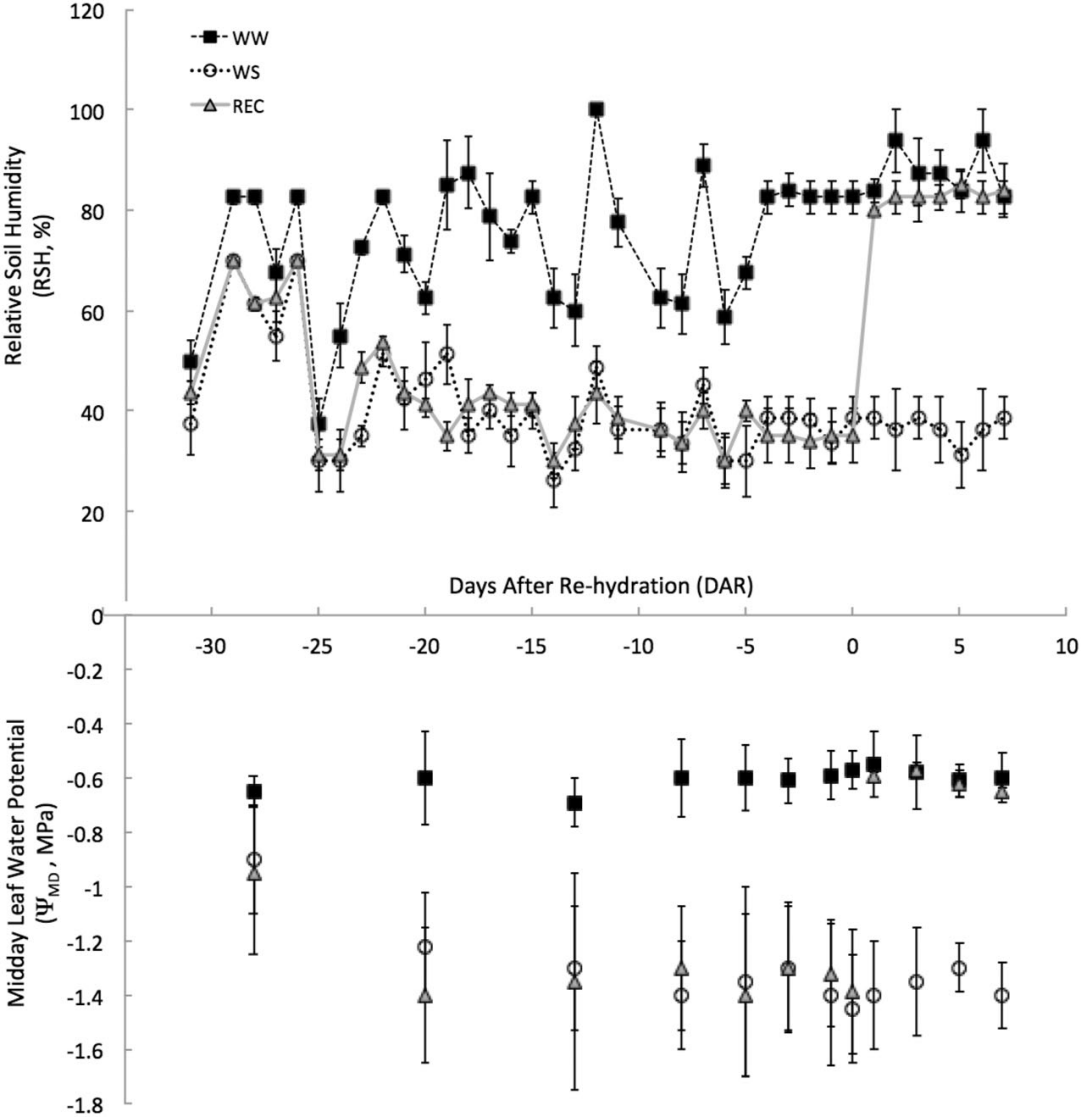
Imposition and maintenance of water deficit levels. Relative soil humidity measured gravimetrically and midday leaf water potential measured by pressure chamber technique on detached leaves. Values are the mean ± SE (*n* = 4).

During the growth season, three irrigation treatments were compared to enable: eight control plants (permanently well irrigated, WW), eight water stress plant (exposed to water stress from the end of July to the end of the experiment, WS) and eight rehydrated plants (exposed to water stress from the end of July to 20 August and well irrigated until the end of the experiment, REC). For each treatment, we randomly selected four plants in plastic pots and four plants in metal pots. A moderate water deficit level (Lavoie‐Lamoureux et al., 2017; Lovisolo et al., 2010; Rienth & Scholasch, 2019) was achieved in approximately 1 week at the beginning of August and maintained until rehydration in REC plants and up to DAR 30 in WS plants. Water stress was achieved and maintained by progressively acting on soil moisture levels, checked gravimetrically approximately every 2 days. The design based on maintaining midday leaf water potential (Ψ_MD_) levels, measured on detached leaves in the plants growing in metal pots by pressure chamber technique, weekly at the beginning of the imposition of water stress and more frequently as *veraison* approached. On REC plants, Ψ_MD_ was restored in 1 day after rehydration, as expected (Lovisolo, Perrone, et al., 2008) (Figure 7).

During the experiment, after rehydration, the relative soil humidity (RSH) in WS pots ranged between 30 and 40% and the pre‐dawn leaf water potential (Ψ_PD_) was also checked (Rodriguez‐Dominguez et al., 2022), with single leaf gas exchange at 10.00 h (Lovisolo et al., 2010) being assessed every 2 days to maintain the designed stress level by replenishing water losses accordingly. In WS plants, Ψ_PD_ was held around −0.18 ± 0.04 MPa, single leaf net CO_2_ assimilation (*A*_leaf_) was around 4.7 ± 2.2 μmol of CO_2_ m^−2^ sec^−1^ and single leaf transpiration (*E*_leaf_) was around 1.2 ± 0.6 mmol of H_2_O m^−2^ sec^−1^, whereas the well‐watered (WW) condition corresponded to RSH > 80%, Ψ_PD_ of −0.05 ± 0.01 MPa, *A*_leaf_ 10.3 ± 2.2 μmol of CO_2_ m^−2^ sec^−1^ and *E*_leaf_ 3.0 ± 0.9 mmol of H_2_O m^−2^ sec^−1^.

The rehydration was carried out on 20 August at 08.00 h, restoring the pot RSH to 80%, similar to that constantly maintained in the control WW plants. In all of the measurements we performed, 20 August was considered as DAR 0 and was designed to coincide with 100% berry *veraison*.

Because a linear correlation was observed between the square leaf maximum width (diameter) and LA of Barbera grapevine (Figure S4), the LA of each plant was estimated *in vivo* by measuring maximum diameter of all leaves according to Vitali et al. (2013). The LA of plants for gas exchange analysis was calculated before and after the measurement campaign (at DAR −7 and at DAR 8) and the LA of plants for carbon labeling was measured at DAR −1, 15 and 30.

At DAR 30, plants in plastic pots were entirely sampled, and leaf, berry and root fresh and dry biomass were quantified. Weight of the berry, production of grapes per plant, number of berries per plant, °Brix of the berries and their total acidity as tartaric acid were assessed.

### Whole plant gas exchange measurements

All of the plants in the metal pots were installed in a multichamber system for continuous gas exchange analysis between whole‐canopy, soil and atmosphere (Patono et al., 2022) (Figure S3). Above‐ground measurement consisted of three custom centrifuge fans (PBN, Mezzago, Italy) blowing atmospheric air into 12 polyethylene (Long Life, Eiffel, Italy) balloons through polyvinyl chloride pipelines. Centrifuge fan velocity was controlled with three inverters (VFD007EL23A; Delta, Taichung City, Taiwan) and air flow incoming into balloons was continuously monitored with hot‐wire anemometers. The temperature inside the balloon was monitored with 12 thermocouples. For soil gas exchange measurements, air flow was supported by three diaphragm pumps (D7 series; Charles Austin, Byfleet, UK) pushing air into metal pots through pneumatic pipelines connected to the pot with pneumatic fittings. Air flow was continuously monitored with mass flow sensors (Top Trak 822; Sierra, Monterey, CA, USA). Air‐volume homogenization was guaranteed by 12 V fans in both balloons and metal pots. Pneumatic probes were positioned to balloons and metal pots outlets and to a centrifuge fan inlet and connected to a CO_2_ and H_2_O gas analyzer (LI‐850; LI‐COR, Lincoln, NE, USA). A manifold with 25 connections with a system of solenoid valves (320 series; Matrix, Ivrea, Italy) made it possible to select the air sampling path.

In detail, there were 12 balloons divided into three modules; into each module, one to two plants per treatment were randomized. Measurements followed a repeated 120‐sec routine including 60 sec of air purging and 60 sec thereafter to determine the mean steady‐state value. The measurements were conducted following the sequence: one reference, six samples, one reference, six samples, one reference. Accordingly, each routine consisted of 1 + 6 + 1 + 6 + 1 = 15 measurements * 120 sec = 30 min. H_2_O and CO_2_ were measured simultaneously. As for reference CO_2_ (ranging between 405 and 425 ppm), there was greater stability than for reference H_2_O (ranging between 13 and 19 mbar), but, by averaging the three references, a stable value was obtained. No gradient was observed when measuring the reference in the 3 modules.

Differential CO_2_ concentration, differential H_2_O concentration and air flow were measured every 6 h in the soil compartment.

All of the electronic instrumentation was connected to a control system (FieldPoint; National Instruments, Austin, TX, USA) and data collection was monitored via an external PC.

The single leaf respiration was measured in the night on replicate leaves with a portable infrared gas analyzer (GFS‐3000, Walz, Germany) to estimate respiration of the whole‐canopy during dark hours (*R*_cd_). Whole‐canopy *A*, *E* and *R*_cd_, as well as below‐ground respiration (*R*_bg_), were calculated following von Caemmerer and Farquhar's equations (von Caemmerer & von Caemmerer & Farquhar, 1981). Plant gas exchange measurements were performed in a period of high pressure and consequent highly‐evapotranspirative atmosphere. Air temperature (*T*), photosynthetic photon flux density (PPFD) and air relative humidity (RH) were monitored and are reported in Figure S5.

### ^13^CO_2_ pulse‐labeling

Three plants for each treatment were labeled with ^13^CO_2_ at DAR 1 within an air‐tight, transparent labeling chamber with an internal volume of 8.4 m^3^ (Intek SRL, Torino, Italy). Before labeling the soil was sealed to minimize diffusion of the labeled CO_2_ into the soil. During labeling, the chamber temperature and RH were set to 28°C and 60%, respectively, whereas natural light was integrated by artificial LED light. The CO_2_ concentration inside the chamber was monitored constantly with a portable infrared gas analysis showing sensitivity to ^13^CO_2_ that was previously determined to correspond to 11% of natural abundance CO_2_. Labeling started at 8.00 h by repeatedly replacing CO_2_ depleted by plant assimilation with 30 atom% ^13^CO_2_ generated through the reaction between 0.6 m NaH^13^CO_3_ (30 atom%) and 4 m sulfuric acid to maintain the CO_2_ concentrations in the chamber between 370 and 420 ppm throughout the labeling period (Figure S1). Plant labeling ended after 4 h after which tissue samples (leaves, berries and primary and secondary roots separated from a soil core) were immediately collected on all nine labeled plants. In addition, three additional pots that remained unlabeled were sampled to provide the natural δ^13^C background of plant compartments. All plant biomass samples were dried at 70°C, weighed, milled prior to δ^13^C analysis. The same sampling procedure was performed at DAR 2, 3, 6, 15 and 30. After the pulse, all 9 + 3 plants were enclosed in air ventilated balloon to reproduce the same condition of plant used for whole plant gas exchange analysis.

### Isotope ratio mass spectrometry measurements

The δ^13^C values and C contents of plant biomass samples were measured by high‐temperature combustion in an elemental analyzer (Vario Isotope Select; Elementar Analysensysteme GmbH, Hanau, Germany) coupled to an isotope ratio mass spectrometer (Isoprime 100; Elementar Analysensysteme GmbH). The δ^13^C‐values (‰) were calibrated relative to the international standard Vienna Pee Dee Belemnite by means of a three‐point calibration using standard reference materials IAEA‐600, IAEA‐603 and IAEA‐CH3. Measurement uncertainty was monitored by repeated measurements of internal laboratory standards and standard reference materials. Precision was determined to be ±0.1‰ based on repeated measurements of calibration standards and internal laboratory standards. Accuracy was determined to be ±0.1‰ on the basis of the difference between the observed and known δ values of check standards and their SD values. The total analytical uncertainty for δ^13^C values was estimated to be ±0.2‰. To estimate ^13^CO_2_ uptake by leaves and translocation to other organs, δ notations were first expressed in atom% and, subsequently, the C content of an organ fraction was multiplied by the ^13^C excess (atom%) of this fraction (with respect to the ^13^C of the unlabeled control), as follows:

$${}^{13}C\;\text{fixed}\;\left(\mathrm{mg}\;\text{plant}{}^{-1}\right)=\frac{\left(\text{atom}\%{}^{13}{\text{C}}_{\text{labeled}}-\text{atom}\%{}^{13}{\text{C}}_{\text{unlabeled}}\right)}{100}∙B∙\frac{C\%}{100}$$

where *B* is the dry weight (DW) of plant biomass compartments (leaf, root or berry) and C% is the percentage of C in the sample. Changes in the total amounts of ^13^C assimilated or delivered in the different plant organs with time were expressed as a percentage of the amount of ^13^C fixed by the leaves at DAR 1 (the labeled ^13^C), assumed to represent the total ^13^C assimilated by the plant during labeling.

To model the total amount of C assimilated at DAR 1 directed to the different C pools and that persisted during the experimental period, we multiplied the integral amount of C assimilated at DAR 1 with the percentage of C partitioning determined from the ^13^CO_2_ pulse labeling:

$$C{\text{allocation}}_{\text{pool},t}\left[\text{mmol}C\right]=\text{Daily}A_{\mathrm{DAR}1}{\left[\text{mmol}\right]}^{*13}C_{\text{pool},t}\left[\%\right]$$

where C allocation_pool, t_ is the total C assimilated at DAR 1 that is allocated and persisted at time *t* in the considered pool. Daily *A*_DAR 1_ is the integral of daily C assimilated at DAR 1. ^13^C_pool, t_ is the percentage of the amount of ^13^C fixed by the leaves at DAR 1 that is present in the C pool at time *t*.

Leaf biomass was calculated using the linear correlation that exist between leaf diameter^2^ and leaf DW (Figure S4) and plant leaf diameters were measured at DAR 0, 6, 15 and 30. Root biomass was dried and weighted at DAR 30. No evidence of root growth was observed for WS plants, whereas there was evidence of root growth for WW and REC plants (root lighter in color with white root tips). In any case, the volume of new roots on the total root volume was negligible. For each plant, a representative portion of the total root system was exanimated, primary and secondary roots were manually separated and weighed, and the relative weight was normalized to the total weight of the root system. Total fruit dry mass was quantified at DAR 30. The average DW of berries sampled at DAR 1, 2 and 3 for each plant was compared with the average DW of berries sampled at DAR 30. An 18% increase of berry DW was observed and it was linearly distributed along the 30 days monitored. No differences in berry DW were observed between treatments.

### Molecular analysis

At 4 h (DAR 0), at 28 h (DAR 1) and at 4 days from rewatering (DAR 4), at 12.00 h, leaf, berry and root samples collected from WW, WS and REC plants (three biological replicates for each treatment) were sampled in liquid nitrogen. Plant materials were ground in liquid nitrogen; 40 mg of leaf and 200 mg of root and berry were used for total RNA extraction with Spectrum Plant Total RNA kit (Sigma‐Aldrich, St Louis, MO, USA). cDNA was synthesized from 1 μg of the total RNA with High Capacity cDNA Reverse Transcription Kit (Life Technologies, Carlsbad, CA, USA). RT‐qPCR analyses were performed as described previously (Chitarra et al., 2017), using the oligonucleotide sets listed in Table S1. Three technical replicates were run for each biological replicate, and the expression of transcripts was quantified after normalization to two housekeeping genes: ubiquitin (VvUBI) and actin (VvACT). One‐way analyses of variance (anova) with treatment as the main factor were performed with spss, version 23.0 (IBM Corp., Armonk, NY, USA). Tukey's honestly significant difference test was applied when anova showed significant differences (*P* < 0.05). The SE of all means was calculated.

## AUTHOR CONTRIBUTIONS

DLP, DS‐P, IP and CL conceptualized and wrote the original draft. DLP, DS‐P, LEA, GI, AFi and IP carried out the experimental part under supervision from GG, DRA, AFe, LC, CL. GG, WC and AFe critically reviewed the draft. All authors read and approved the final version of the manuscript submitted for publication.

## CONFLICT OF INTEREST

The authors declare no conflict of interest.

### DATA AVAILABILITY

All relevant data can be found within the manuscript and its supporting materials.

## Supporting information


**Table S1.** List of the oligonucleotides used in the present study.
**Figure S1**. ^13^CO_2_ pulse of nine grapevine plants under climate‐controlled conditions at DAR 1 in the labeling chamber.
**Figures S2**. Transcripts of key genes of sugar metabolism. Relative expression level of (a) sucrose synthase (*VvSusy*), cell wall invertase (*VvcwINV*), threalose 6‐phosphate phosphatase (*VvTPP*) and starch synthase (*VvSTA*) and (b) hexose transporter 3 (*VvHT3*), Sugar Will Eventually be Exported Transporter 10 (*VvSWEET10*), hexose transporter 6 (*VvHT6*) and vacuolar invertase 2 (*VvGIN2*) genes in leaf, root and berry tissues sampled from WW, WS and REC plants at DAR 0 and DAR1.
**Figure S3**. Multichamber system for continuous gas exchange analysis.
**Figure S4**. Leaf area (LA) index of *Vitis vinifera cv* Barbera grafted onto *Vitis riparia* × *Vitis berlandieri* 420A rootstocks.
**Figure S5**. Environmental check during the whole‐plant gas exchange analysis. (a) air temperature (T), (b) photosynthetic photon flux density (PPFD) and (c) air relative humidity (RH).
