# Supplementary material for: Photosynthetic recovery in drought‐rehydrated grapevines is associated with high demand from the sinks, maximizing the fruit‐oriented performance
Source: Plant J. 2022 Oct 28;112(4):1098–111. doi: 10.1111/tpj.16000 (PMC9828513; doi:10.1111/tpj.16000)
Supplement: Supplementary file 1 — Table S1. List of the oligonucleotides used in the present study. Figure S1. 13CO2 pulse of nine grapevine plants under climate‐controlled conditions at DAR 1 in the labeling chamber. Figures S2. Transcripts of key genes of sugar metabolism. Relative expression level of (a) sucrose synthase (VvSusy), cell wall invertase (VvcwINV), threalose 6‐phosphate phosphatase (VvTPP) and starch synthase (VvSTA) and (b) hexose transporter 3 (VvHT3), Sugar Will Eventually be Exported Transporter 10 (VvSWEET10), hexose transporter 6 (VvHT6) and vacuolar invertase 2 (VvGIN2) genes in leaf, root and berry tissues sampled from WW, WS and REC plants at DAR 0 and DAR1. Figure S3. Multichamber system for continuous gas exchange analysis. Figure S4. Leaf area (LA) index of Vitis vinifera cv Barbera grafted onto Vitis riparia × Vitis berlandieri 420A rootstocks. Figure S5. Environmental check during the whole‐plant gas exchange analysis. (a) air temperature (T), (b) photosynthetic photon flux density (PPFD) and (c) air relative humidity (RH). [file TPJ-112-1098-s001.docx]

Table S1.

| **Target (Gene ID)** | ***Gene abbreviation*** | **Gene Description** | **Primer** | **Primer sequences 5'-3'** | **References** |
| --- | --- | --- | --- | --- | --- |
| VIT_11s0016g00470 | *VvSuSy* | Sucrose synthase | Forward | TGTTAAGGCTCCTGGATTTCAATTA | Prezelj et al., 2016 |
|  |  |  | Reverse | AGCCAAATCTTGGCAAGCA |  |
| VIT_09s0002g02320 | *VvcwINV* | Cell wall apoplastic invertase | Forward | AGGAGGTGGAAAGGTTTGCATA | Ferrero et al., 2018 |
|  |  |  | Reverse | TGGGCTTCACCGTCAATAGC |  |
| VIT_00s0304g00080 | *VvTPP* | Trehalose-6-phosphate phosphatase | Forward | TCCATCCCAGGAGCAAGTGT | Gambino et al., 2012 |
|  |  |  | Reverse | CACAGCGGTAATGCACAGAGA |  |
| VIT_02s0025g02790 | *VvSTA* | Starch synthase | Forward | GGCGACTCTGACTGCTTCTCA | Gambino et al., 2012 |
|  |  |  | Reverse | CCTGGGTGCCGTTGACAT |  |
| VIT_11s0149g00050 | *VvHT3* | Hexose transporter 3 | Forward | AGTACGACAACCAAGGGCTACAG | Gambino et al., 2012 |
|  |  |  | Reverse | GAGGTCAAGCCCGCAAGATA |  |
| VIT_17s0000g00830 | *VvSWEET10* | Sugar Will Eventually be Exported Transporter | Forward | TATCTGCGGATTCGGTTCCA | Prezelj et al., 2016 |
|  |  |  | Reverse | ACGCTTAGCGAGAACACGAGAC |  |
| VIT_18s0122g00850 | *VvHT6* | Hexose transporter 6 | Forward | TTCTTGAAGGTGCCCGAGAC | Pagliarani et al., 2019 |
|  |  |  | Reverse | AGTAACCTGCCTTGCTCCAAC |  |
| VIT_02s0154g00090 | *VvGIN2* | Vacuolar invertase 2 | Forward | CCAACCAAGGCGATCTATG | Prezelj et al., 2016 |
|  |  |  | Reverse | TTGAGGCAGTGATGCTGG |  |
| VIT_04s0044g00580 | *VvACT* | Actin | Forward | TCCGTTCTCAGAGATCAACAA | Gambino et al., 2012 |
|  |  |  | Reverse | ACTCTCTCATCTCAAGATATTCTATGG |  |
| VIT_16s0098g01190 | *VvUBI* | Ubiquitin | Forward | TCTGAGGCTTCGTGGTGGTA | Gambino et al., 2012 |
|  |  |  | Reverse | AGGCGTGCATAACATTTGCG |  |

Supplementary Table 1. List of the oligonucleotides used in this study.

Figure S1.

Supplementary Figure 1. ^13^CO_2_ pulse of 9 grapevine plants under climate-controlled condition at DAR 1 in the labelling chamber. 1) Electrical panel for temperature and humidity control, 2) nine grapevines in plastic pots equipped with connection pipes and fittings for balloon setup, 3) External PC connected to the IRGA for labelling chamber CO_2_ concentration monitoring, 4) injection system explained in the white panel 5) artificial LED light for natural light integration.

Figure S2a.





Figure S2b.





Supplementary Figure 2. Transcripts of key genes of sugar metabolism. Relative expression level of (a) sucrose synthase (*VvSusy*), cell wall invertase (*VvcwINV*), threalose-6-phosphate phosphatase (*VvTPP*), starch synthase (*VvSTA*), and (b) hexose transporter 3 (*VvHT3*), Sugar Will Eventually be Exported Transporter 10 (*VvSWEET10*), hexose transporter 6 (*VvHT6*) and vacuolar invertase 2 (*VvGIN2*) genes in leaf, root and berry tissues sampled from WW, WS and REC plants at DAR 0 and DAR1, as determined by qRT-PCR signals normalized to actin (*VvACT*) and ubiquitin (*VvUBI*) transcripts. Data are presented as the mean ±SE of three biological and technical replicates. Gene IDs and oligonucleotides used for each gene are indicated in the Table S1. Different lowercase letters above the bars indicate significant differences according to a *post hoc* Tukey's test (p ≤ 0.05).

Figure S3.


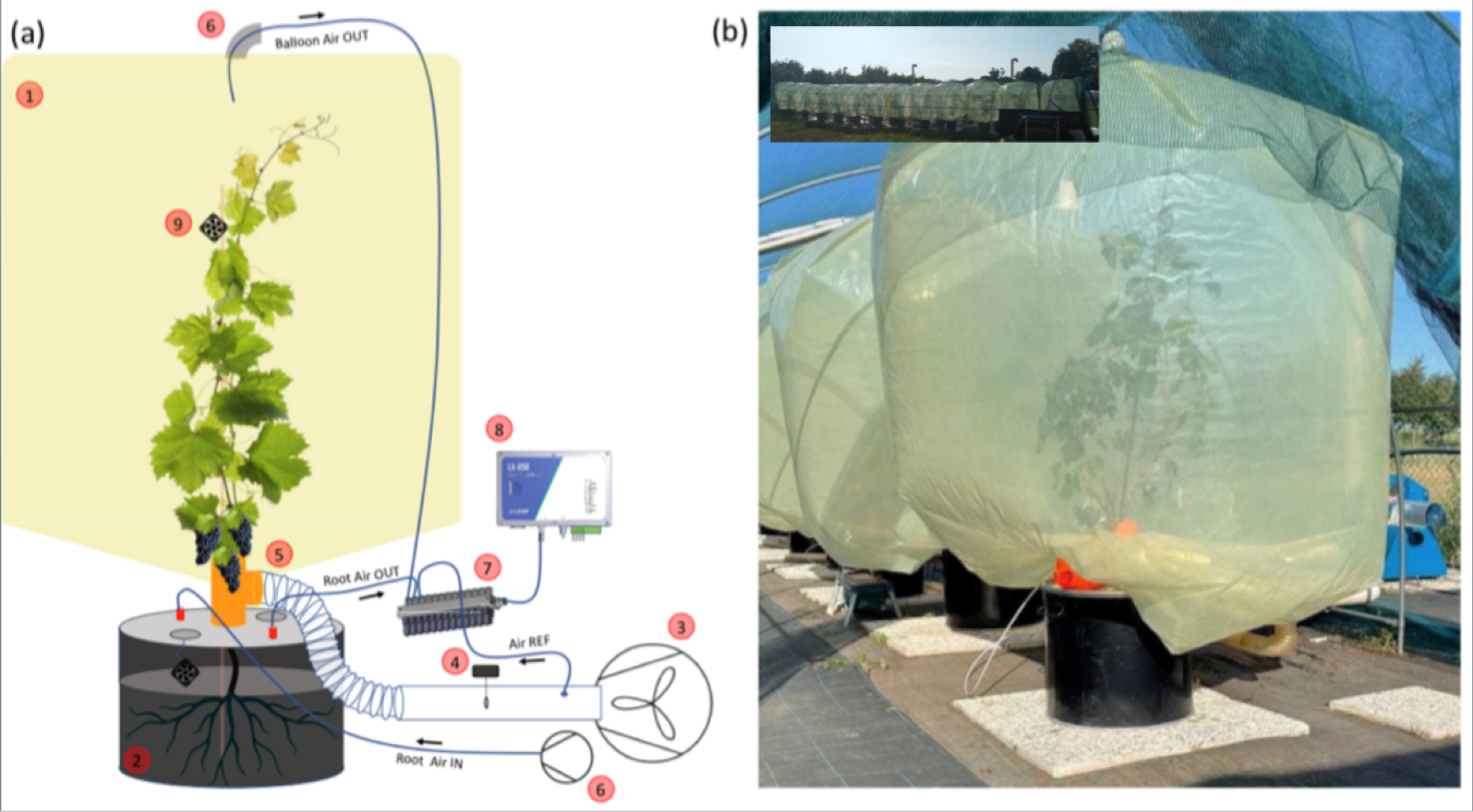


Supplementary Figure 3. Multi-chamber system for continuous gas exchange analysis. (a) schematic representation of multi-chamber system: 1) PE balloon, 2) Custom made metallic pot, 3) centrifuge fan, 4) hot-wire anemometer, 5) T junction for balloon air inlet, 6) diaphragm pump, 7) manifold with solenoid valves, 8) IRGA, 9) 12V DC fan. (b) Multi-chamber system during measurements. In the upper panel a 12-multi-chamber platform.

Figure S4.

Supplementary Figure 4. Linear correlation between leaf area (LA) and leaf diameter^2^ (d^2^) and linear correlation between leaf dry weight (DW) and d^2^ of *Vitis vinifera* *cv* Barbera grafted onto *Vitis riparia* × *Vitis berlandieri* 420A rootstocks.

Figure S5.

Supplementary Figure 5. Environmental check during the whole-plant gas exchange analysis. (a) air temperature, T; (b) photosynthetic photon flux density, PPFD; (c) air relative humidity, RH.
